# Spatio-temporal patterns and determinants of persistent catastrophic health expenditure in China: evidence from the China Family Panel Studies

**DOI:** 10.3389/fpubh.2025.1658120

**Published:** 2025-12-04

**Authors:** Man-ci Zhou, Zi-xuan Chen, Ke-ming Fan, Jing Guo, Xiao-hua Wang, Shuang Ma

**Affiliations:** 1 School of Management, Beijing University of Chinese Medicine, Beijing, China; 2 Vanke School of Public Health, Tsinghua University, Beijing, China; 3 School of Health Policy and Management, Chinese Academy of Medical Sciences & Peking Union Medical College, Beijing, China; 4 School of Public Health, Peking University, Beijing, China; 5 School of Government, Beijing Normal University, Beijing, China

**Keywords:** catastrophic health expenditure, China Family Panel Studies, influencing factors, spatio-temporal patterns, out-of-pocket, universal health coverage

## Abstract

**Background:**

The escalating out-of-pocket medical expenses pose a significant threat to the financial stability of households worldwide. Identifying persistent catastrophic health expenditure (CHE) is imperative for alleviating medical-related economic difficulties. This study comprehensively investigated the spatio-temporal dynamics and key determinants of persistent CHE in China across the 2012–2020 period.

**Methods:**

This longitudinal quantitative study leveraged data from five waves of the China Family Panel Studies (CFPS), conducted biennially from 2012 to 2020. Variables pertaining to the incidence, depth, and duration of CHE were scrutinized, with covariates informed by the Andersen health service utilization model, including predisposing, enabling, and need factors. Spatio-temporal patterns were analyzed through spatial autocorrelation analysis. Tobit regression was employed to analyze CHE depth, whereas a Generalized Estimating Equation model was used to investigate factors influencing the duration of CHE.

**Results:**

The analytical sample comprised a balanced panel of 2,544 households, yielding a total of 12,720 observations. Although the incidence of CHE decreased from 16.27 to 13.88%, there was a persistent upward trajectory in the intensity of CHE, which rose from 0.026 in 2012 to 0.069 in 2020. The proportion of households experiencing persistent CHE saw a marked increase, escalating from 0% in 2012 to 14.32% in 2020. Rural households demonstrated a higher incidence of CHE compared to urban households, and significant regional variations in persistent CHE were evident. The spatial clustering of persistent CHE experienced a dynamic transition from aggregation to dispersion and then to a partial recovery during 2012–2020. Factors such as poor self-assessed health status (OR = 1.015, 95% CI 0.011 to 0.194; *p* < 0.05), a greater number of family dependents (OR = 1.047, 0.040 to 0.053; *p* < 0.05), the presence of chronic diseases (OR = 1.013, 0.006 to 0.021; *p* < 0.05), and hospitalization (OR = 1.069, 0.053 to 0.081; *p* < 0.05) were significantly associated with an elevated risk of persistent CHE.

**Conclusion:**

Financial protection against persistent CHE remains a challenge in China, particularly for rural households, low-income groups, families with chronic diseases, and regions with underdeveloped economies. Policy must prioritize enhancing insurance benefit packages, establishing persistent CHE-linked targeted assistance, and implementing region-specific strategies to address the root causes of health-induced poverty.

## Introduction

The escalation of out-of-pocket (OOP) medical expenditures and barriers to healthcare access exert a notably detrimental impact on households’ financial resilience (1). This phenomenon stems from either abrupt or incremental medical cost burdens, coupled with transient or enduring income disruptions (2). It manifests as a self-reinforcing cycle where poverty and morbidity become inextricably intertwined, perpetuating a sustained economic strain on affected families and individuals (3).

Globally, tackling financial burdens is a priority, as evidenced by the Sustainable Development Goals (SDGs). To fully depict the healthcare economic burden and financial vulnerability risks associated with illness in a country (or region), the concept and core indicator of “Catastrophic Health Expenditure” (CHE) are widely used internationally (4). CHE refers to OOP medical expenses not covered by health insurance, constituting ≥40% of total household expenditure excluding food spending (5, 6).

The two mainstream methods for measuring CHE are the budget share approach and the capacity-to-pay approach (7). The budget share approach takes total household expenditure or income as the denominator, with conventional thresholds of 10% or 25%. In the capacity-to-pay approach, the denominator is household expenditure after subtracting basic needs, typically with a 40% threshold (alternative thresholds like 25% are also used). Using different thresholds or denominators on the same data can significantly alter the incidence and distribution of CHE, thereby impacting equity assessments and policy prioritization (8, 9).

Existing evidence has identified several key drivers of CHE. First, socio-economic, and demographic factors revealed that low income, low education, rural residence, old age, and comorbidities are generally associated with a higher risk of CHE (10). Second, health status and disease burden played a critical role. A study utilizing CHARLS data identified hospital admissions and outpatient visits as the primary drivers of CHE incidence (11). Third, the structure of the medical insurance system and patterns of healthcare utilization also exert a profound influence (12). Evidence suggested that systemic deficiencies, such as limited reimbursement lists, flawed deductible and copayment structures, and inadequate coverage for outpatient and chronic services, exacerbated the risk of households facing financial hardship (13, 14). Given the significant impact of CHE on poverty vulnerability, long-term assessment and strategic planning are essential for preventing illness-induced poverty and its recurrence (15). However, this long-term perspective remains underdeveloped in existing literature. Most studies rely on a single threshold and static snapshots, making it difficult to link findings to regional payment systems or supply structures (16, 17). Consequently, there was still a lack of studies analyzing dynamic analysis of persistent CHE and the inter-provincial disparities therein. Furthermore, research perspectives have predominantly centered on individual economic income levels, emphasizing a singular dimension of poverty, while the assessment of healthcare costs at the household level is relatively unexplored (18, 19).

Over the past decade, China’s healthcare reform has shown notable progress, particularly in effective measures to control medical expenses, resulting in a decrease in personal health expenditures from 40.4% in 2008 to 27.0% in 2022 (20). In 2012, the overall coverage rate of basic health insurance reached over 95%. However, according to the World Health Organization’s data, over one billion people globally faced CHE in 2019, with a global incidence rate of 13.5% (21). The incidence of CHE in China varied significantly across different studies. Yuan Q et al. found that China experienced an average incidence rate of CHE at 25.5% from 2018 to 2020 (22). A systematic review and meta-analysis reported a notable increase in CHE incidence in China from 11.7% in 2000–2008 to 28.0% in 2017–2020 (23). Additionally, some studies indicated an overall incidence of CHE around 17% and demonstrating a declining trend (24, 25). The higher incidence of CHE in China compared to the global average indicated that some households still bear a heavy economic burden due to illness. Therefore, studying the current status and influencing factors of CHE in China is of great significance for alleviating medical economic difficulties and promoting health equity.

This study systematically identified spatio-temporal patterns to understand inter-regional CHE inequality and estimate the trends and determinants of persistent CHE in China, spanning from 2012 to 2020. The China Family Panel Studies (CFPS) constitutes a nearly nationwide, large-scale, longitudinal social survey designed to facilitate research on a broad spectrum of contemporary Chinese social phenomena (26). Leveraging data from CFPS, a comprehensive and large-scale nationwide social tracking survey, findings from this study may have implications for setting future healthcare policy priorities in China, and may offer valuable insights for other countries in the Western-Pacific region, thereby achieving the SDGs by 2030.

## Methods

### Study design

This longitudinal quantitative study employed a balanced panel design to investigate the spatio-temporal patterns and determinants of persistent CHE in China from 2012 to 2020.

### Setting

This study utilized data from five waves of the CFPS, conducted in 2012, 2014, 2016, 2018, and 2020. Launched in 2010 by the Institute of Social Science Survey at Peking University, the CFPS is a biennial nationwide household survey frequently used for estimating CHE and conducting related research (25, 27, 28). The baseline sample covers 25 provinces, autonomous regions, and municipalities, representing 94.5% of Mainland China’s total population. The sample is derived through a multi-stage probability sampling method that employs implicit stratification. CFPS subsamples were hierarchically drawn, starting at the county level, then the village level, and finally the household level. Detailed sampling protocols are available at www.isss.edu.cn/cfps/. Participants underwent biennial follow-ups, resulting in a total of six national survey periods to date (26). To maintain ethical standards, the Peking University Biomedical Ethics Review Committee granted approval (Approval number: IRB00001052-14010). Respondents were provided with a detailed statement outlining the study’s purpose, and their consent was obtained before data collection commenced.

### Participants

The baseline cohort of the CFPS in 2010 included 14,960 households and 42,590 individuals. No new households were added to the panel subsequently. The success-tracking rates for the follow-up periods in 2012, 2014, 2016, 2018, and 2020 were 85, 89, 89, 86, and 89%, respectively. This study used the individual characteristics of household heads (designated as financial respondents) as representative of the family’s individual characteristics within the sample. To address potential selection bias from panel attrition, we utilized a balanced panel of households that participated in all five waves. After excluding observations with missing data, our analytic sample consisted of a balanced panel involving 2,544 households, comprising a total of 12,720 observations.

### Variables and measurement

#### The incidence of CHE

The incidence of CHE was assessed as the proportion of households whose OOP for health care constituted at least 40% of a household’s capacity to pay (29, 30). Household capacity to pay was defined as their annual total household expenditure after deducting annual food consumption costs within a year and the OOP payment was an estimation of aggregate annual health costs paid by the household with a 12 month recall period from the household head (31, 32). All economic variables, including total household expenditure, food consumption, and OOP, were measured at the household level and represent annual amount in the CFPS survey.

#### The persistent depth and duration of CHE

This study introduced a temporal dimension into index calculations, by formulating a function to assess the duration and depth of CHE, aiming to provide a more accurate and comprehensive measurement of its long-term impact on households. The duration of CHE is defined as the maximum number of consecutive periods during which a household experiences CHE across the five periods tracked in the study, with values ranging from 0 to 5 periods. Following the duration analysis method proposed by Foster et al. (33), households encountering CHE for 2 or more consecutive periods are categorized as facing persistent CHE. The persistent depth of CHE, also referred to as the deprived share of persistent CHE, ranges from 0 to 1. This study specifically defined the persistent depth of CHE as the proportion of total OOP health care expenses incurred by households experiencing persistent CHE, exceeding certain thresholds relative to the household’s financial capacity.

### Confounding variables

The selection of covariates was guided by the Andersen health service utilization model, providing a comprehensive framework for analysis (34). According to this model, health service utilization is influenced by predisposing, enabling, and need variables.

Predisposing variables primarily include household size, household residence (urban or rural), gender of the household head (male or female), age of the household head, marital status of the household head (living alone, married or partnered), job classification (unemployed, agricultural worker, and non-agricultural worker), years of education of the household head, and the number of family dependents (members aged >over 60 years or under<6 years). Enabling variables were measured using three indicators: type of health insurance held by the household head, geographic location (northeast, west, middle, central eastern areas), and socioeconomic group. The sample was categorized into five groups based on equivalent household capacity to pay, ranging from the most deprived 20% to the most affluent 20%. Health insurance options included the urban employee basic medical insurance scheme (UEBMI), the urban resident basic medical insurance scheme (URBMI), or new cooperative medical scheme (NCMS), and being uninsured (9). Regarding need variables, the study considered members with chronic diseases, self-rated health (ranging from worst to best), and hospitalization (yes or no).

### Statistical methods

Descriptive statistics were used to succinctly outline the characteristics of the study sample. Categorical data were presented through frequency and proportion, whereas continuous data were summarized using mean and standard deviation. This study explored the spatial and temporal distribution characteristics of CHE incidence, depth, and duration among sample families from 2012 to 2020. Additionally, this study employed global and local Moran’s I indices to examine the spatial patterns of the incidence, depth, and duration of CHE across 31 provinces in China. The Global Moran’s I index estimated the spatial agglomeration and divergence, with a value ranging from −1 to 1 (35). Global Moran’s I > 0 signifies positive spatial agglomeration, I < 0 indicates negative spatial correlation, and I = 0 denotes a random spatial distribution. The local Indicator of Spatial Association (LISA) is designed to further examine the spatial association between a local unit and its neighboring units. Local Moran’s I > 0 means positive correlation between an observation and its neighbors (high values surrounded by high values, or low by low). I < 0 indicates negative spatial dependence (higher-lower or lower-higher clusters). The statistical significance of the global and local Moran’s I indices is assessed using the z-value (36). The Tobit model is a regression model designed for situations where the dependent variable is censored or truncated.

Given the threshold limitations within the study context, a significant number of instances in the depth of CHE were observed with values of zero. Therefore, Tobit regression analysis was employed as a methodological approach to comprehensively examine the determinants of CHE depth. Furthermore, this study analyzed five-wave longitudinal data collected from the same households, which resulted in within-subject correlation due to the repeated observations. While conventional models for cross-sectional data (e.g., logistic regression) cannot account for this autocorrelation, the Generalized Estimating Equations (GEE) model addresses it by incorporating an appropriate working correlation matrix (37). Consequently, a GEE model was constructed under a Gaussian distribution to discern the factors influencing the duration of CHE. Statistical significance was set at *p* < 0.05, and 95% confidence intervals were reported. All statistical analyses were executed using Stata 16.0, and spatial analyses were conducted using ArcGIS Pro software.

## Results

### Descriptive analysis

Table 1 outlines the comprehensive characteristics of the surveyed households from the years 2012, 2014, 2016, 2018, and 2020, with a particular focus on the 2,544 households that consistently participated in all surveys. The average household size decreased from a mean of 4.007 members in 2012 to 3.816 members in 2020. The majority of household heads resided in rural areas (with the percentage declining from 75.28 to 71.05%), and most lived with a spouse or partner, decreasing from 92.02 to 86.79%. The proportion of householders working in agriculture saw a slight decrease from 41.97% in 2012 to 39.81% in 2020, while the number of family dependents increased from 0.85 to 1.01 over the same period. The number of households with UEBMI rose from 409 in 2012 to 478 in 2020, whereas those with URBMI or NCMS decreased from 1956 to 1879. The prevalence of chronic diseases among household members increased from 0.372 to 0.401, and the percentage of householders who perceived themselves to be in good health decreased from 78.774% in 2012 to 75.977% in 2020.

**Table 1:** Characteristics of surveyed households in 2012, 2014, 2016, 2018, and 2020 (*N* = 2,544).

Characteristics	2012		2014		2016		2018		2020	
	Number (Mean)	% (SD)	Number (Mean)	% (SD)	Number (Mean)	% (SD)	Number (Mean)	% (SD)	Number (Mean)	% (SD)
*Predisposing variables*										
Household size	4.007	1.699	3.947	1.755	3.859	1.813	3.811	1.846	3.816	1.908
Household residence										
Rural	1915	75.28	1937	76.17	1847	72.63	1786	70.99	1777	71.05
Urban	629	24.73	606	23.83	696	27.37	730	29.01	724	28.95
Household head gender										
Male	1,435	56.41	1,421	55.86	1,368	53.77	1,393	54.76	1,500	58.96
Female	1,109	43.59	1,123	44.14	1,176	46.23	1,151	45.24	1,044	41.04
Household head age	49.43	12.27	49.67	12.82	49.82	14.00	52.02	13.72	52.60	13.92
Household head marital status										
Living alone	203	7.98	241	9.47	305	11.99	291	11.52	331	13.21
Married or partnered	2,341	92.02	2,303	90.53	2,239	88.01	2,235	88.48	2,174	86.79
Job classification										
Unemployed	969	44.74	507	20.01	545	21.58	549	22.58	542	21.71
Agricultural worker	909	41.97	1,237	48.82	1,125	44.55	1,035	42.58	994	39.81
Non-agricultural worker	288	13.30	790	31.18	855	33.86	847	34.84	961	38.49
Education years	6.717	4.68	7.589	4.359	7.794	4.437	7.889	4.437	8.293	4.519
Number of family dependents	0.85	0.94	0.99	0.97	1.00	0.97	1.06	0.97	1.01	0.93
*Enabling variables*										
Location										
Northeast	387	15.21	388	15.25	387	15.21	388	15.25	384	15.12
Western	794	31.21	783	30.78	772	30.35	778	30.58	771	30.35
Central	627	24.65	619	24.33	605	23.78	613	24.10	600	23.62
Eastern	736	28.93	754	29.64	780	30.66	765	30.07	785	30.91
Socioeconomic group										
Quintile I (most deprived)	508	19.97	508	19.97	507	19.97	508	19.97	508	19.97
Quintile II	509	20.01	509	20.01	508	20.01	509	20.01	510	20.05
Quintile III	509	20.01	509	20.01	507	20.01	509	20.01	508	19.97
Quintile IV	509	20.01	509	20.01	508	20.01	509	20.01	509	20.01
Quintile V (most affluent)	509	20.01	509	20.01	508	20.01	509	20.01	509	20.01
Insurance type										
Not insured	190	7.47	153	6.01	159	6.25	162	6.37	234	9.20
UEBMI	409	16.08	460	18.08	457	17.96	488	19.18	478	18.79
URBMI/NCMS	1956	76.89	1969	77.40	1956	76.89	1928	75.79	1879	73.86
*Need variables*										
Member with chronic disease	0.372	0.613	0.512	0.690	0.483	0.695	0.506	0.682	0.401	0.624
Health need: self-rated health										
Bad	163	6.41	247	9.71	259	10.18	266	10.46	288	11.38
Fair	377	14.82	409	16.08	348	13.68	287	11.29	320	12.64
Good	886	34.83	977	38.40	938	36.87	1,061	41.74	1,128	44.57
Better	521	20.48	410	16.12	505	19.85	383	15.07	336	13.28
Best	597	23.47	501	19.69	494	19.42	545	21.44	459	18.14
Hospitalization										
No	2,272	89.45	2,225	87.77	2,199	86.85	2,111	83.54	2,159	86.71
Yes	268	10.55	310	12.23	333	13.15	416	16.46	331	13.29

SD, standard deviation; UEBMI, Urban Employee Basic Medical Insurance scheme; URBMI, Urban Resident Basic Medical Insurance scheme; NCMS, New Cooperative Medical Scheme.

### Spatio-temporal patterns of persistent catastrophic health expenditure

**Figure 1:**
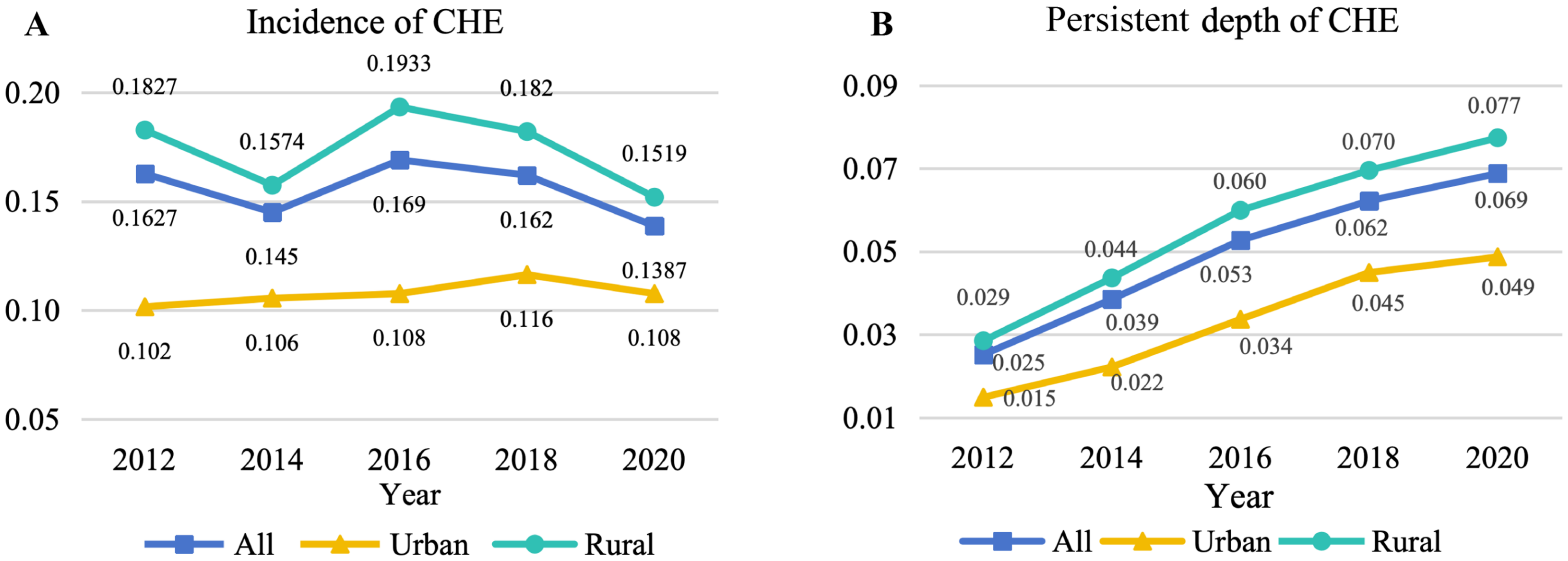
Level and change in CHE incidence and persistent depth (using the 40% threshold), 2012–2020. **(A)** Incidence of CHE. **(B)** Persistent depth of CHE.

Figure 1 illustrates the levels and variations in the depth and incidence of persistent CHE in China from 2012 to 2020. The incidence of CHE has demonstrated a fluctuating pattern, characterized by a sequence of “decrease, increase, decrease” over the years, with recorded rates of 16.27, 14.50, 16.98, 16.23, and 13.88% from 2012 to 2020, respectively. Although the fluctuations in CHE among urban households have not been statistically significant, there has been a marginal increase from 10.17% in 2012 to 10.77% in 2020. The overall trend for rural households closely mirrors the national average, showing a decline from 18.27% in 2012 to 15.19% in 2020. The persistent depth of CHE has consistently followed an ascending trajectory, increasing from 0.025 in 2012 to 0.069 in 2020. At the urban level, the persistent depth of CHE has experienced an elevation from 0.015 in 2012 to 0.049. Similarly, at the rural level, the persistent depth of CHE has decreased from 0.029 in 2012 to 0.077 in 2020.

**Figure 2:**
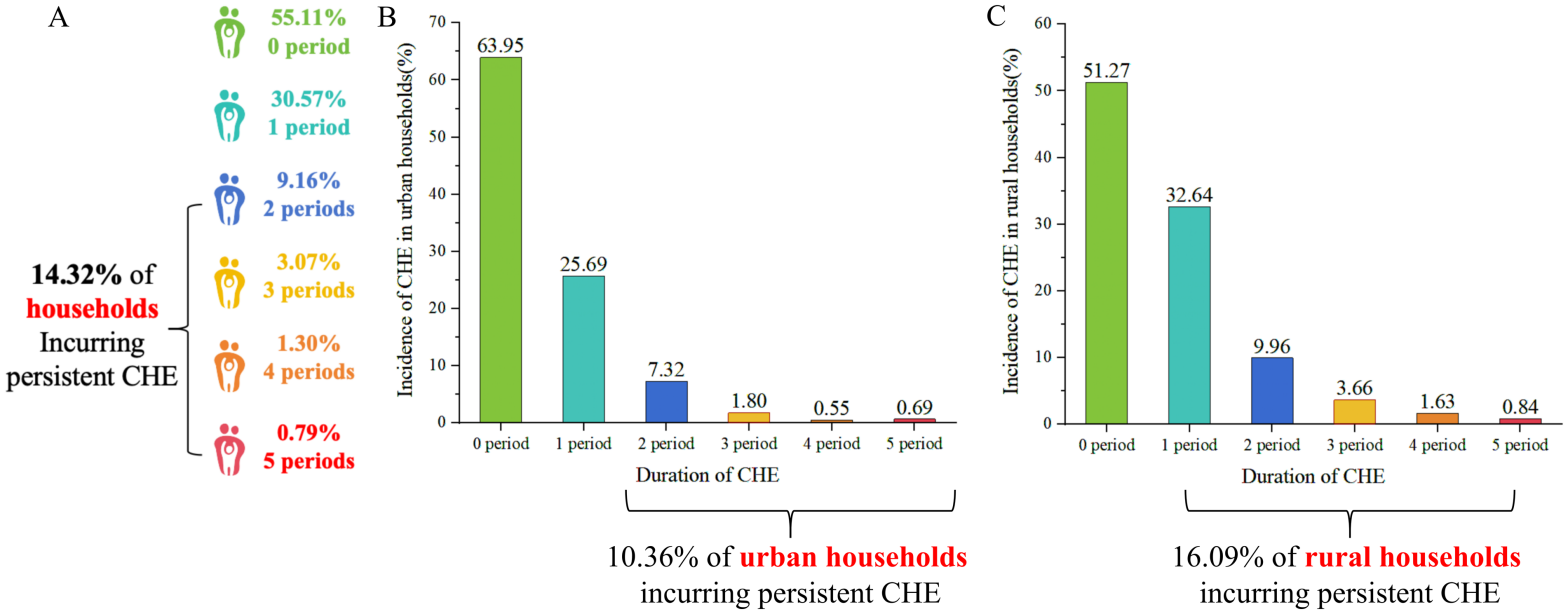
Incidence of CHE across different durations in sample households in China, 2020. **(A)** Incidence of CHE across different durations in total households. **(B)** Incidence of CHE across different durations in urban households. **(C)** Incidence of CHE across different durations in rural households.

Figure 2 depicts the distribution of households experiencing different durations of CHE in 2020 as a percentage of the total number of households. The prevalence of households affected by persistent CHE has increased notably, with an average annual growth rate of 2.39% over a five-year period. In 2020, the proportion of households affected by persistent CHE reached 14.32% in 2020. When comparing urban and rural households, the percentages were 10.36 and 16.09%, respectively, in 2020. This disparity indicates that rural families are more vulnerable to the prolonged effects of CHE than urban counterparts.

**Figure 3:**
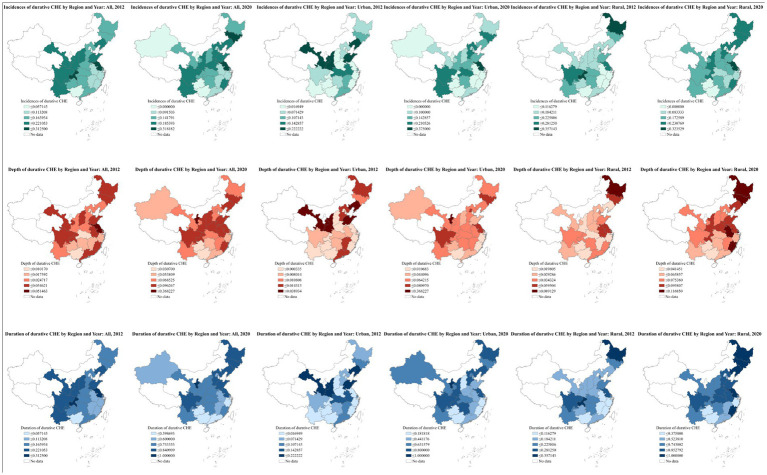
Indicators of persistent CHE (using the 40% threshold) across Chinese provinces, stratified by rural and urban areas. The blank parts indicate missing data. The provinces with missing data in 2012 included Hong Kong, Macau, Taiwan, Xinjiang, Tibet, Qinghai, Inner Mongolia, Ningxia, and Hainan. The provinces with missing data in 2020 included Hong Kong, Macau, Taiwan, Tibet, Qinghai, Inner Mongolia, Ningxia, and Hainan.

To visually represent the spatial patterns of CHE, this study created spatial distribution maps of CHE measurement indices (Figure 3). At the national level, CHE indices in 2012 showed a general trend of increasing from east to west. By 2020, however, this trend had changed to an increase from south to north. In urban areas, spatial disparities have diminished, and by 2020, a pattern of increasing indices from east to west became apparent. In rural areas, initially, the higher CHE indices were concentrated in the northeast, but by 2020, there was a shift towards a continuous increase in indices from south to north.

The global spatial autocorrelation analysis in Table 2 revealed that the spatial clustering of CHE duration was statistically significant only in 2012 (Moran’s I = 0.294, *p* < 0.05). From 2012 to 2016, Moran’s I of persistent CHE showed a significant decrease, changing from positive to negative values. Subsequently, from 2016 to 2020, Moran’s I rebounded from negative to weakly positive values but remained substantially lower than the initial level observed in 2012.

**Table 2:** Global Moran index of persistent CHE, 2012–2020.

Year	Depth of CHE		Duration of CHE	
	Moran’s index	*Z*	Moran’s index	*Z*
2012	0.207	1.575	0.294*	2.126
2014	0.025	0.991	0.137	1.448
2016	−0.071	−0.55	−0.219	−1.448
2018	−0.075	−0.566	−0.228	−1.426
2020	0.042	0.325	0.194	1.464

*p < 0.1, **p < 0.05, ***p < 0.01.

**Figure 4:**
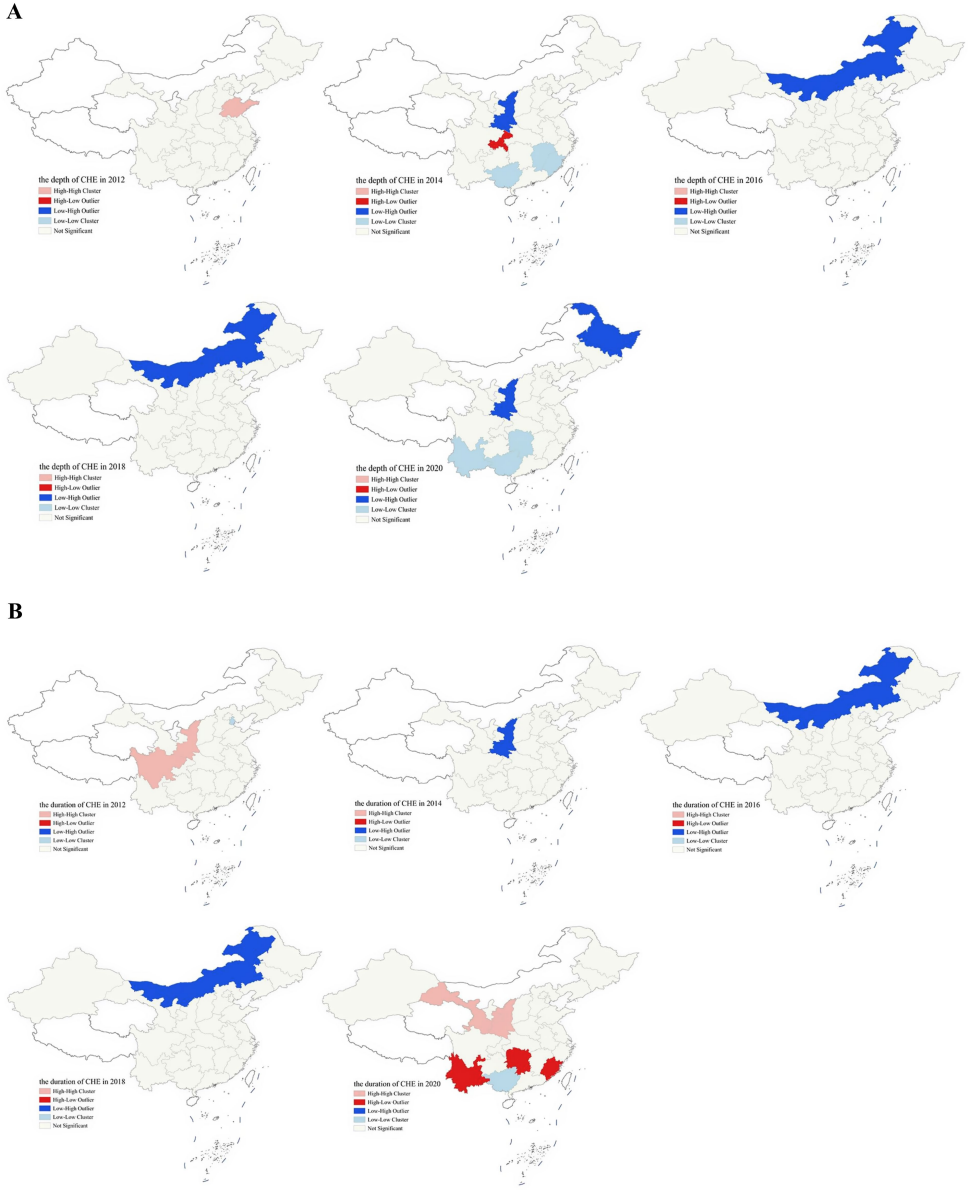
LISA of persistent CHE in China, 2012–2020. **(A)** Depth of CHE. **(B)** Duration of CHE.

To more intuitively illustrate the local spatial correlation of persistent CHE, this study plotted LISA maps for the depth and duration of CHE across the years 2012, 2014, 2016, 2018, and 2020 (Figure 4). As for depth of CHE, in 2012, a high-high cluster was observed in Shandong province. By 2014, however, the spatial pattern had diversified: Guangxi, Jiangxi, and Fujian formed low-low clusters, Chongqing emerged as a high-low outlier, and Shaanxi was identified as a low-high outlier. Inner Mongolia was consistently recognized as a low-high clustering region in 2016 and 2018. In 2020, low-low clusters were identified in Guangxi, Yunnan, and Hunan, and high-low outliers were observed in Heilongjiang and Shaanxi. Concerning the duration of CHE, Shanxi was identified as a low-high cluster in 2012, while a significant high-high cluster was observed in Gansu and Ningxia. In 2016 and 2018, the spatial pattern shifted notably, with Inner Mongolia being classified as an LH outlier. By 2020, Gansu and Shaanxi formed a high-high cluster; Guangxi was identified as a low-low cluster; and Fujian, Yunnan, and Hunan were characterized as high-low clustering pattern.

### Determinants of persistent catastrophic health expenditure

Table 3 presents factors influencing persistent CHE using Tobit and GEE models. Tobit regression indicated that household size was associated with a reduction in the depth of CHE (OR = 0.973), whereas protective factors included higher education levels of household heads (OR = 0.996), marital status (OR = 0.955), employment in agriculture or non-agriculture sectors (OR = 0.969 and 0.944), residence in the eastern region (OR = 0.977), and possession of UEBMI insurance (OR = 0.969). In contrast, factors contributing to an increased depth of CHE were the presence of family dependents (OR = 1.047) and chronic disease (OR = 1.013).

**Table 3:** Determinants of persistent CHE in Tobit model and GEE model.

Characteristics	Depth of CHE			Duration of CHE		
	Coef (SE)	OR	95%CI	Coef (SE)	OR	95%C
*Predisposing variables*						
Household size	−0.027*** (0.002)	0.973	[−0.030, −0.023]	−0.041*** (0.004)	0.960	[−0.049, −0.033]
Household residence (rural)						
Urban	−0.062*** (0.007)	0.940	[−0.077, −0.047]	−0.063*** (0.016)	0.939	[−0.095, −0.032]
Household head gender (male)						
Female	−0.005 (0.005)	0.995	[−0.016, 0.005]	−0.009 (0.011)	0.991	[−0.029, 0.012]
Household head age	0.0007** (0.0002)	1.001	[0.0002, 0.0012]	0.003*** (0.001)	1.003	[0.002, 0.004]
Household head marital status (Living alone)						
Married or partnered	−0.046*** (0.008)	0.955	[−0.061, −0.030]	−0.121*** (0.018)	0.886	[−0.156, −0.086]
Job classification (unemployed)						
Agricultural worker	−0.032*** (0.007)	0.969	[−0.046, −0.018]	−0.043*** (0.013)	0.958	[−0.068, −0.018]
Non-agricultural worker	−0.058*** (0.008)	0.944	[−0.074, −0.042]	−0.08*** (0.014)	0.923	[−0.108, −0.053]
Household head education years	−0.004*** (0.0007)	0.996	[−0.006, −0.003]	−0.005** (0.002)	0.995	[−0.008, −0.002]
Number of family dependents	0.046*** (0.003)	1.047	[0.040, 0.053]	0.056*** (0.006)	1.057	[0.043, 0.068]
*Enabling variables*						
Location (Northeast)						
Western	0.005 (0.008)	1.005	[−0.011, 0.021]	−0.024 (0.037)	0.976	[−0.096, 0.048]
Central	−0.012 (0.008)	0.988	[−0.028, 0.004]	−0.051 (0.037)	0.950	[−0.124, 0.023]
Eastern	−0.023** (0.008)	0.977	[−0.038, −0.007]	−0.096** (0.035)	0.908	[−0.166, −0.027]
Socioeconomic group (Quintile I)						
Quintile II	−0.012 (0.008)	0.988	[−0.027, 0.003]	−0.023 (0.013)	0.978	[−0.048, 0.002]
Quintile III	−0.008 (0.008)	0.992	[−0.023, 0.008]	−0.039** (0.014)	0.962	[−0.066, −0.013]
Quintile IV	−0.014 (0.008)	0.986	[−0.030, 0.002]	−0.031* (0.014)	0.970	[−0.059, −0.003]
Quintile V (most affluent)	0.013 (0.009)	1.013	[−0.004, 0.030]	−0.023 (0.015)	0.978	[−0.052, 0.007]
Household head insurance type (URBMI/NCMS)						
Not insured	0.003 (0.010)	1.003	[−0.016, 0.022]	−0.007 (0.016)	0.993	[−0.038, 0.024]
UEBMI	−0.031** (0.009)	0.969	[−0.053, −0.008]	−0.007 (0.016)	0.993	[−0.038, 0.024]
*Need variables*						
Member with chronic disease	0.013*** (0.004)	1.013	[0.006, 0.021]	0.015* (0.007)	1.015	[0.002, 0.028]
Health need: self-rated health	0.015*** (0.002)	1.015	[0.011, 0.194]	0.01* (0.004)	1.010	[0.002, 0.018]
Hospitalization (no)						
Yes	0.067 (0.007)	1.069	[0.053, 0.081]	0.079*** (0.012)	1.082	[0.055, 0.103]
Year (2012)						
2014	0.075*** (0.009)	1.078	[0.057, 0.093]	0.176*** (0.012)	1.192	[0.153, 0.199]
2016	0.126*** (0.009)	1.134	[0.108, 0.143]	0.312*** (0.012)	1.366	[0.289, 0.336]
2018	0.153*** (0.009)	1.165	[0.135, 0.170]	0.43*** (0.012)	1.538	[0.406, 0.455]
2020	0.183*** (0.009)	1.201	[0.165, 0.201]	0.528***(0.012)	1.695	[0.503, 0.552]
(Constant)	−0.129*** (0.024)	0.879	[−0.176, −0.083]	0.333*** (0.055)	1.396	[0.225, 0.442]

(A) Depth of CHE. (B) Duration of CHE. *p < 0.1, **p < 0.05, ***p < 0.01. SE, standard error; OR, odds ratio.

The GEE model was constructed with the duration of CHE as the dependent variable. It revealed that older household heads (OR = 1.003), a greater number of family dependents (OR = 1.057), chronic disease (OR = 1.015), poorer health status (OR = 1.010), and recent hospitalization (OR = 1.082) were correlated with a longer duration of CHE. Larger household size (OR = 0.960) and higher education levels of household heads (OR = 0.995) acted as protective factors. Urban residence and marriage were associated with reduced sensitivity to the duration of CHE (OR = 0.939 and 0.886), while households with heads employed in agricultural (OR = 0.958) and non-agricultural (OR = 0.923) sectors experienced shorter durations compared to those with no employment. Furthermore, families in Quintiles III (OR = 0.962) and IV (OR = 0.970) experienced shorter durations compared to those in the most deprived families.

### Sensitivity analysis

Sensitivity analyses for the 10 and 25% thresholds indicated an increase in the severity and persistence of CHE from 2012 to 2020 (*p* < 0.05), mirroring a similar trend observed when the threshold was set at 40%. The associations between the duration and depth of CHE and the independent variables remained unchanged. However, the statistical significance of certain factors varied (see Supplementary Table 1 for further details).

## Discussion

To our knowledge, this was the first longitudinal quantitative study systematically exploring the spatio-temporal patterns and determinants of persistent CHE in China during 2012–2020 using five waves of CFPS data. Our findings indicated that while the incidence of CHE decreased from 2012 to 2020, its depth and duration increased. Spatially, the clustering of persistent CHE transitioned from aggregation to dispersion and then partial recovery. Key risk factors included poor health status, chronic diseases, and hospitalization, while urban residence, higher education, and employment were protective factors.

### The temporal distribution characteristics of CHE incidence, depth, and duration

This study indicated that the incidence of CHE decreased from 16.27% in 2012 to 13.88% in 2020. This observed decline may be attributed to the synergistic impact of economic growth and various policy interventions, particularly the expansion of universal health insurance coverage and increased subsidies for vulnerable groups (28). In line with prior research utilizing comparable CHE measurements, our findings broadly align with these studies, indicating an overall incidence of CHE around 14% and demonstrating a declining trend (24, 25). However, the number of households impacted by persistent CHE saw a significant rise, with an average annual increase of 2.39% over a five-year period. Additionally, a consistent upward trend in the persistent depth of CHE, increasing from 0.025 in 2012 to 0.069 in 2020, underscoring a growing financial crisis for households. Families facing conditions like cancer or uremia may not be excluded from the medical insurance system but can still enter a cycle of “treatment-debt-retreatment,” leading to chronic financial exhaustion. The cause may lie in the design of medical insurance schemes. Despite expanded coverage, medical insurance schemes with high OOP ratios, low reimbursement caps, and restrictive drug formularies offer inadequate protection against the long-term financial impact of major chronic diseases (38).

Given the pronounced disparities in economic levels, social security systems, and health resources between urban and rural areas in China, the incidence, persistent depth, and duration of CHE in rural households exceed those in urban households (24, 27, 28). This disparity stemmed from deeper, systemic vulnerabilities that can be analyzed across three key dimensions. First, the concentration of high-quality medical resources in urban areas forces rural residents to bear higher indirect costs (e.g., transportation, accommodation) for non-local treatment, exacerbating their direct financial burden. Second, despite the integration of urban and rural resident health insurance, substantive gaps remain compared to UEBMI in terms of reimbursement rates, coverage ceilings, and drug formularies, resulting in shallower financial protection for rural residents. Third, rural households typically have more singular income sources, weaker savings, and a less robust social safety net, inherently lowering their financial resilience (24, 39).

### The spatial distribution characteristics of CHE incidence, depth, and duration

Our regional investigation has revealed significant inter-regional disparities in the duration of CHE. At the national level, CHE indices in 2020 indicated a shift from the southern to the northern regions. This uneven distribution of CHE across China was affected by differences in financial contributions, treatment packages, and reimbursement rates among various health insurance policies (40, 41). Further global autocorrelation analysis revealed that the spatial pattern of persistent CHE in China underwent a general shift from “clustered” to “relatively dispersed” between 2012 and 2020, which was consistent with findings from others (42). The Global Moran’s I for CHE duration was statistically significant only in 2012 (*p* < 0.05). The lack of statistical significance in Moran’s I for CHE duration during 2014–2020 can be attributed to three factors. First, following the expansion of universal health insurance coverage and targeted increases in reimbursement rates for rural and low-income groups, the regional gap in financial protection against CHE narrowed. Second, the sustained migration of rural working-age populations to urban areas altered the demographic structure of high-risk groups for persistent CHE. Third, the increasing prevalence of chronic diseases shifted the disease burden from historically high-prevalence regions to more geographically dispersed areas. Local Indicators of Spatial Association (LISA) further supported this transition. Our results identified high-high clusters of CHE duration in Gansu and Ningxia provinces in both 2012 and 2020. This spatial persistence stems from regions’ fragile economic foundations, worsened population aging from young adult outmigration, and an underdeveloped healthcare insurance system unable to provide robust safeguards. Therefore, it is crucial to improve health insurance coverage, support capacity building in county and primary medical institutions, and create local jobs to retain young talent. In contrast, low-low clusters were mainly observed in regions such as Guangxi, potentially due to broader health insurance coverage and policy support, as well as more severe CHE issues in neighboring provinces. Due to its resource-based economy, central fiscal transfers, and lighter demographic burden, Inner Mongolia consistently showed a low-high clustering pattern. For such areas, fostering cross-regional medical cooperation and addressing internal development disparities are recommended. Yunnan and Hunan were high-low clusters in CHE duration in 2020 but low-low in depth, meaning households faced mild shocks but lacked recovery capacity. Addressing this requires strengthening chronic disease management and enhancing household economic resilience.

### Protective factors for CHE

The GEE and Tobit models have illuminated that larger households, those situated in urban and eastern regions, and households with higher levels of education, employed members, and married household heads are associated with a decreased risk of incurring persistent CHE. The correlation between enhanced education levels and lower incidences of CHE suggests a plausible link to increased awareness of disease prevention and superior healthcare knowledge among families with higher education (43). In larger families with married household heads, the distribution of economic risks is apparent, indicative of a greater capacity to afford healthcare costs (25, 43). Additionally, the stability in income and social security for employed household heads further contributes to mitigating the risk of CHE (28). These factors primarily guarded against household financial distress and facilitate rapid recovery by enhancing ex-ante prevention and immediate payment capacity, as well as by relying on the household’s long-term financial resilience, social capital, and continuous risk-sharing capacity.

Compared to URBMI/NRCMS, UEBMI demonstrated a significant reduction in the duration and depth of CHE among the sampled families. This finding aligns consistently with existing literature, which indicates that UEBMI are capable of protecting some households from the impact of CHE (23, 44). UEBMI predominantly serves urban employed individuals, who generally enjoy more stable incomes and superior social security coverage. Conversely, the URBMI/NRCMS extends coverage to both urban and rural residents, some of whom may have lower incomes and less comprehensive social security coverage, thereby potentially increasing their vulnerability to CHE (20). Furthermore, UEBMI provides higher reimbursement rates, thus facilitating a more effective reduction of the financial burden of household medical expenses compared to URBMI/NRCMS (45).

### Risk factors for CHE

Families experiencing overlapping vulnerabilities in their economy, health, and social conditions were more susceptible to falling into the cycle of poverty and illness (46, 47). Our study identified several risk factors for chronic household economic burden, including that households with a higher number of family dependents, members with chronic diseases, those living in low economic conditions and rural areas, those utilizing hospitalization services, and those with a low-level health status of the household head. For families managing chronic illness or disability, healthcare costs represent a persistent financial drain. If economic hardship forces them to compromise treatment, it can precipitate a cycle of deteriorating health and escalating medical expenses. To cope, households often resort to strategies like selling productive assets or taking on informal loans, which severely erode their long-term capacity to generate income and escape poverty (48). This situation is exacerbated when the ill individual or their caregiver is the primary earner, leading to a sustained loss of household income. The concurrent shock of rising expenses and falling income constitutes a core mechanism that deepens and perpetuates CHE. Consequently, it is crucial to implement effective, targeted interventions to disrupt this cycle.

### Limitations

Several factors contribute to the limitations of the findings in this study. Firstly, it’s important to note that OOP medical expenses recorded in the CFPS survey only encompass direct medical costs, excluding non-medical expenses such as transportation, accommodation, and income losses. This exclusion may result in an underestimation of both the frequency and severity of CHE. Consequently, future studies should focus on the mechanisms that influence these factors. Secondly, considering that this study relies on self-reported health information provided by household heads, there is a possibility of reporting errors. Such errors could potentially affect the accuracy and reliability of the findings.

### Policy implication

The findings underscore the need for a more equitable and sustainable health financing system to mitigate the persistence of CHE. First, the adoption of a progressive universalism framework, which prioritizes low-income and high-risk populations, is central to achieving equitable universal health coverage and optimizing financial protection (49). Second, expanding reimbursement rates and outpatient and chronic-disease coverage, while dynamically adjusting benefit packages and national reimbursement drug lists, would reduce the persistent financial burden on households with chronic conditions or rapidly deteriorating health status. Meanwhile, narrowing institutional and regional disparities requires integrating urban–rural insurance schemes, raising pooling levels, and harmonizing benefit packages between the UEBMI and URBMI/NRCMS systems (50). Finally, establishing a targeted assistance and early-warning mechanism, underpinned by persistent monitoring of CHE, is instrumental in breaking the “illness–poverty” cycle. Cross-sectoral collaboration among medical insurance, social welfare, and financial services can facilitate this shift. By enabling stratified assistance and low-interest medical loans, coupled with chronic-disease management and primary-care follow-up, the system can transition from ex-post financial compensation to a paradigm of proactive, pre-emptive prevention (49, 51).

## Conclusion

In conclusion, this study has comprehensively analyzed the eight-year development of CHE in China from 2012 and 2020. During this period, although the incidence of CHE decreased from 16.27% in 2012 to 13.88% in 2020, there was a consistent upward trend in both the depth and duration of CHE. The depth increased from 0.026 in 2012 to 0.069 in 2020, and the duration increased from 0.163 to 0.672 over the same period.

Rural households experience a higher incidence of CHE compared to urban households. Regional disparities in the depth of CHE are evident across China’s eastern, central, and western regions. Factors such as low economic status, poor self-rated health, a higher number of family dependents, the presence of chronic diseases, and hospitalization increase the risk of experiencing CHE. Therefore, policies must evolve from broad coverage to deep financial protection, focusing on reforming insurance schemes, providing targeted assistance to high-risk households, and implementing tailored regional interventions to effectively interrupt the cycle of poverty and illness.

## Data Availability

The datasets presented in this study can be found in online repositories. The names of the repository/repositories and accession number(s) can be found in the article/Supplementary material.
