# Correction: Spatio-temporal patterns and determinants of persistent catastrophic health expenditure in China: evidence from the China Family Panel Studies

**DOI:** 10.3389/fpubh.2025.1761344

**Published:** 2025-12-19

**Authors:** Man-ci Zhou, Zi-xuan Chen, Ke-ming Fan, Jing Guo, Xiao-hua Wang, Shuang Ma

**Affiliations:** 1 School of Management, Beijing University of Chinese Medicine, Beijing, China; 2 Vanke School of Public Health, Tsinghua University, Beijing, China; 3 School of Health Policy and Management, Chinese Academy of Medical Sciences & Peking Union Medical College, Beijing, China; 4 School of Public Health, Peking University, Beijing, China; 5 School of Government, Beijing Normal University, Beijing, China

**Keywords:** catastrophic health expenditure, China Family Panel Studies, influencing factors, spatio-temporal patterns, out-of-pocket, universal health coverage

In the published article, there was a mistake in the **Funding** statement. The second fund name was displayed as “grant 2024-JYB-PY-004 from the Basic Research Business Expenses for the year 2023”. The correct statement is “Grant 2024-JYB-PY-004 from the Fundamental Research Funds for the Central Universities for the year 2024”.

The original article has been updated.

