# Supplementary material for: Spatio-temporal patterns and determinants of persistent catastrophic health expenditure in China: evidence from the China Family Panel Studies
Source: Front Public Health. 2025 Dec 4;13:1658120. doi: 10.3389/fpubh.2025.1658120 (PMC12711740; doi:10.3389/fpubh.2025.1658120)
Supplement: Supplementary file 1 [file Table_1.DOCX]

**Supplementary Materials**

**Supplemental Table 1: Determinants of persistent CHE (using 25% threshold) in Tobit model and GEE model**

| Characteristics | Depth of CHE | | Duration of CHE | |
| --- | --- | --- | --- | --- |
|  | Coef (SE) | OR | Coef (SE) | OR |
| **Predisposing variables** |  |  |  |  |
| Household size | -0.022***  (0.001) | 0.978 | -0.053***  (0.005) | 0.948 |
| Household Residence (rural) |  |  |  |  |
| Urban | -0.053***  (0.006) | 0.948 | -0.050*  (0.021) | 0.951 |
| Household head gender (male) |  |  |  |  |
| Female | 0.004  (0.005) | 1.004 | 0.012  (0.014) | 1.012 |
| Household head age | 0.0007***  (0.0002) | 1.001 | 0.004***  (0.0008) | 1.004 |
| Household head marital status (Living alone) |  |  |  |  |
| Married or partnered | -0.036***  (0.007) | 0.965 | -0.130***  (0.024) | 0.878 |
| Job classification (No job) |  |  |  |  |
| Agriculture | -0.035**  (0.006) | 0.966 | -0.054***  (0.002) | 0.947 |
| Non-agriculture | -0.052***  (0.007) | 0.949 | -0.099***  (0.018) | 0.906 |
| Household head education years | -0.004***  (0.0005) | 0.996 | -0.005*  (0.002) | 0.995 |
| Number of family dependents | 0.043***  (0.003) | 1.044 | 0.083***  (0.008) | 1.087 |
| **Enabling variables** |  |  |  |  |
| Location (Northeast) |  |  |  |  |
| Western | 0.0004  (0.007) | 1.000 | -0.050  (0.046) | 0.951 |
| Middle | -0.015*  (0.007) | 0.985 | -0.135**  (0.047) | 0.874 |
| Eastern | -0.024***  (0.007) | 0.976 | -0.181***  (0.045) | 0.834 |
| Socioeconomic group (Quintile I) |  |  |  |  |
| Quintile II | -0.009  (0.006) | 0.991 | -0.049**  (0.016) | 0.952 |
| Quintile III | -0.007  (0.007) | 0.993 | -0.081***  (0.017) | 0.922 |
| Quintile IV | -0.012  (0.007) | 0.988 | -0.101***  (0.018) | 0.904 |
| Quintile V (most affluent) | 0.006  (0.007) | 1.006 | -0.086***  (0.019) | 0.918 |
| Household head insurance type (URBMI/NCMS) |  |  |  |  |
| Not insured | 0.006  (0.009) | 1.006 | 0.009  (0.022) | 1.009 |
| UEBMI | -0.030***  (0.007) | 0.970 | -0.059**  (0.021) | 0.943 |
| **Need variables** |  |  |  |  |
| Member with chronic disease | 0.015***  (0.003) | 1.015 | 0.023**  (0.008) | 1.023 |
| Health need: self-rated health | 0.015***  (0.002) | 1.015 | 0.012*  (0.005) | 1.012 |
| Hospitalization (no) |  |  |  |  |
| Yes | 0.062***  (0.006) | 1.064 | 0.087***  (0.016) | 1.091 |
| Year (2012) |  |  |  |  |
| 2014 | 0.067***  (0.007) | 1.069 | 0.310***  (0.015) | 1.363 |
| 2016 | 0.106***  (0.007) | 1.112 | 0.523***  (0.015) | 1.687 |
| 2018 | 0.127***  (0.007) | 1.135 | 0.706***  (0.015) | 2.026 |
| 2020 | 0.154***  (0.007) | 1.166 | 0.885***  (0.016) | 2.423 |
| (Constant) | -0.047**  (0.018) | 0.954 | 0.531***  (0.068) | 1.701 |

* p < 0.05; ** p < 0.01; *** p < 0.001. OR, odds ratio. SE, Standard Error.
